# The British Journals: Surgery

**Published:** 1841-07

**Authors:** 


### SURGERY.

*On the Employment of Threads of Caoutchouc for Satures.* By Thomas Nunneley, Esq., Surgeon to the Leeds General Eye and Ear Infirmary.

More than twelve months since I was induced to try several substances, in the hope of discovering one which should be free from the objections incident to the ordinary ligatures; this, I think, I have succeeded in, or, at least, I have found one which certainly possesses many advantages over the filamentous material now commonly employed; it is that substance which is now applied to so many purposes in the arts—caoutchouc, drawn out into fine strings; these threads I have hitherto obtained by removing the fibrous envelope of cotton or silk, with which the Indian-rubber itself is surrounded in the elastic web, at present so commonly employed for bandages, not being able to procure in Leeds the elastic strings alone, but in London, or where the fabric is manufactured, there could be no difficulty in obtaining them.

The advantages which it possesses as a ligature are, the much longer time it remains without producing irritation of any kind; being elastic, it holds the divided parts in contact with much less stretching, in a more natural manner, and, at the same time, it keeps up an equal degree of tension; for if the parts swell, the ligature gives way in proportion to the pressure; on the contrary, do they contract, so also does the ligature, and an equable approximation is maintained. From being smooth and unirritating, it excites so little disturbance that a greater number of ligatures may be introduced, and thus the parts may be more accurately adjusted to each other; moreover, by not inducing ulceration, the scars left after its withdrawal are less than those produced by silk, are advantages not to be lost sight of in operations about the face and neck.

I need scarcely add, that the very qualities which render the caoutchouc thread preferable for sutures for wounds, totally unfit it as ligatures for vessels, where a sharp unelastic string is necessary for first dividing the inner coats, and subsequently retaining them in contact. *Lancet. March* 13, 1841.

*Case of Neuralgia Facialis, cured by Operation.* By T. T. LAMBERT, Surgeon, Hull.

E. L., a female, aged sixty-three, had for several years been affected with a most painful condition of the nerves of one side of the face, coming on in paroxysms of the utmost severity, mostly towards night. At various times she had tried most of the usual remedies, without experiencing anything but temporary alleviation. Under these circumstances, as the branches of the facial nerve seemed to be principally affected, the pain shooting across the cheek down towards the lower jaw, and occasionally upwards towards the temple, I proposed the division of the facial nerve in the parotid gland, after its exit from the foramen. To this the poor woman gladly consented, and I proceeded to perform the operation by making an incision betwixt the mastoid process and the lobe of the ear. My next incision brought me in contact with a nerve, which I supposed, from its situation, to be the auriculus magnus nerve, one of the ascending branches of the cervical plexus. On raising this nerve with my forceps, a state of tension seemed to be produced upon all the nerves of the face which were affected with the pain, and the patient exclaimed that was the place from whence all her pain proceeded. Under these circumstances, as the operation for the division of this nerve is trivial compared with the division of the facial, and as it was just at hand, I determined to try the effects of its division; and the result was most satisfactory: the woman passed a delightful night, and enjoyed some good sleep. The next day she had a little pain, and from that time to the present she has been perfectly well, except having, on one occasion, a very trifling degree of pain. Four months have now elapsed since the performance of this operation, and during this interval the patient has been exposed to the intense inclemency of the recent winter. I am not one of those who would pay too much attention to a solitary case; yet, as a single fact, this seems to me to be important, inasmuch as the means employed have effectually cured, for four months at least, and I hope permanently, a very severe case of neuralgic pain.

*London Medical Gazette. March* 12, 1841.

---

*On the Section of Muscles in Spinal Curvature.* By ROBERT HUNTER, M.D. Professor of Anatomy, Andersonian University, Glasgow.

I BELIEVE I was the first in this country to perform the subcutaneous section of the dorsal muscles for the cure of lateral curvature of the spine, and as I have now had some experience upon this subject I shall lay the result of my observations briefly before your readers. In no instance has the operation of itself produced a cure; but in all the cases on which I have operated, with one exception, it manifestly placed the patient in a more favorable state for the performance of a cure. The operation itself appears to me to effect no more than to take off, either in part or whole, the power of muscles that are interested in maintaining the curvature, and thus placing the spine in a condition to be more easily influenced by mechanical and physiological causes.

The cases which have been treated by me have all been of long standing, none less than seven years, and some ten, sixteen, and twenty years, and all with considerable torsion and gibbosity, as well as lateral curvature. In fact, they were cases which were either considered absolutely hopeless, or on which medical skill had been exerted for years without the slightest benefit. In some instances the section of the muscles was instantaneously followed by an obvious improvement in the state and appearance of the back; in other instances I could discover no change whatever.

I perform the subcutaneous section of the dorsal muscles at four different places of the back. 1st, I weaken the tension of the deepest seated layer of muscles—that formed by the multifidus spinæ—by dividing the thickest part of that muscle, as it lies comparatively superficially upon the dorsum of the sacrum, opposite the posterior superior spinous process of the ilium. 2d and 3d, I remove the tension of the middle layer of spinal muscles, that formed by the longissimus dorsi and sacro-lumbalis, by cutting these muscles across, sometimes in the lumbar region and sometimes in the costal region, according to the circumstances of the case; but more frequently in the lumbar region, near the origin of these muscles. 4th, To destroy the tension of the flat and more superficial muscles, I divide these muscles by a longitudinal incision, close to the spinous processes of the vertebræ, at the place where the tension of these muscles appears to be the greatest. In one instance I cut through, with considerable effect, the latissimus dorsi, at the side of the chest, and consequently at some distance from the spine. The muscle crossed the contracted and concave side of the trunk, and appeared to be accessory in huddling in the ribs of that side. When the patient attempted to elongate that side, a cord, as thick as the little finger, was seen stretching from the crest of the ilium to the scapula; as soon as this rigid cord of muscle was cut through, the ribs became less huddled together, and the side could be elongated to a much greater degree, and the spine materially affected.

The cutting of the dorsal muscles is only the first, though an important step in the treatment of spinal deformities. The means that are afterwards employed in conducting such cases to a successful issue are both mechanical and physiological. The first consists in the application of pressure, made in various ways and by various means to assist in the gradual return of the parts to their natural places, and the second, without which the first would be useless and unavailing, consists in infusing power into the muscles which have become weak or dormant from disease, by simply calling these muscles frequently and in various combinations into action. *London Medical Gazette. March* 26, 1841.

---

*On the use of Splints in Chorea.* By G. Southam, Surgeon, Salford.

I was led to try the experiment of confining the affected limb in splints, having frequently observed, in those instances where the convulsions had continued in spite of all remedial means, that if the patient's attention be directed to the deranged muscles, as for instance when in the hand and arm, causing him to grasp the sides of a chair, the motions comparatively cease; and this, when frequently repeated, proved a useful auxiliary in removing the disease. Upwards of two years ago a case came under my notice where the convulsions had existed in the hand and arm for six months, though a variety of means had been adopted for their removal: believing them to depend on habit, from the control the patient had over the motions, and his apparently excellent health, I had recourse to the splints. For the first few days the twitchings, from their violence, caused some difficulty in keeping the splints firm, but they soon diminished in force, and in three weeks had entirely disappeared. I have tried the same treatment in four other cases. In three, after attending to the bowels, I applied the splints, and continued them until a cure was established, which occupied in none more than a month. The convulsions were confined to one of the upper extremities, with slight dragging of the leg of the same side; but as the symptoms left the arm, the power over the leg returned. The other case occurred in a youth fifteen years of age. The convulsions were more severe, and rendered it extremely difficult to keep the splints firm with an ordinary bandage: to obviate this inconvenience, I used the apparatus recommended by Velpeau for fractures, with the addition of small splints to keep the limb extended and at rest during the consolidation of the apparatus. This was removed in a week to allow of exercise; and the severity of the convulsions being subdued, a common bandage and splints were again used. Though the convulsions affected both arm and leg, and splints were only applied to the former, the boy had recovered the use of both extremities in six weeks.

*London Medical Gazette. May* 7, 1841.

